# Episodic Migraine and Older Adults

**DOI:** 10.1007/s11916-022-01029-7

**Published:** 2022-04-06

**Authors:** Nina Riggins, Annika Ehrlich

**Affiliations:** 1 University of California San Diego, 4510 Executive Drive # 325, San Diego, CA 92121 USA; 2 UCSD Medical School, San Diego, CA USA; 3 UCSF School of Nursing, San Francisco, CA USA

**Keywords:** Headache, Migraine, Episodic, Elderly, Older, Geriatric

## Abstract

**Purpose of Review:**

Migraine is and continues to be a significant medical issue in older adults. Migraine can have different characteristics in older adults and specific diagnostic and treatment considerations need to be applied when managing headache and migraine in this population, which is increasing in both size and diversity. Contrary to widely held beliefs, migraine may not improve in older women following menopause and can have new onset in older age. The purpose of this review is to give an update on the diagnosis and treatment of episodic migraine in older adults.

**Recent Findings:**

As the population ages, migraine in older adults will become a more significant public health issue. Migraine in older adults can present with different clinical symptoms than in a younger population and is primarily a diagnosis of exclusion in older adults. Migraine treatment considerations for older adults should include comorbidities and medication interactions. Recent findings suggest there are medications that should be avoided when treating seniors with migraine.

**Summary:**

The purpose of this review is to give an update on the most important aspects regarding the diagnosis and treatment of headache and migraine in older adults. In addition, recommendations will be made concerning medications that need careful consideration when prescribing to seniors.

## Introduction

Many headache neurologists frequently asked if migraine will improve after menopause, or if it is possible to develop new onset of migraine in older age? This review endeavors to answer these questions. Research shows that migraine can be a significant issue in older adults, and migraine might not improve after menopause. In addition, migraine can have different characteristics in older adults and specific diagnostic and treatment considerations need to be applied when managing headache and migraine in seniors. While studies show that multiple treatments can be used safely, opioids should be avoided whenever possible. Newer classes of medications such as gepants (small molecule calcitonin gene-related peptide (CGRP) receptor antagonists) and neuromodulation devices are appropriate for older adults.

## Migraine Can Happen at Any Age

Migraine onset can happen at any age [1, 2]. Less people report their first migraine episode after 60 years of age than earlier in life [1, 3], and migraine is certainly most active prior to 50 years of age [1, 4, 5]. However, in a targeted systematic review, an important point was made that the elderly and disabled population have a relatively high burden of migraine (16.4%), which is almost as high (17.9%) as that found in the 18–44-year-old age group. This same review also highlighted that the prevalence of self-reported migraine (1 out of 6 people in the USA) has now remained stable for over 19 years. Additionally, in the USA, 1 out of 5 women over a 3‐month period self-reported migraine or severe headache. Thus, migraine is an important public health issue with a significant burden on patients, including historically disadvantaged population groups [1, 4, 5].

Migraine has a 10% prevalence in older adults on an annual basis and is the second most common headache disorder after tension headache in older adults [1, 2, 6].

An informative study was published [7] reviewing headache in seniors above the age of 65 who sought medical attention for memory issues or cognitive decline. Severe cognitive impairment or dementia were exclusion criteria. In that study, 1237 patients (51.6% women), with a mean age of 75.6 years (SD = 6.9) were screened and it was reported that 24.4% of patients had headaches. An additional finding from this study was that probable migraine (13.8% of all patients) was the most common type of headache reported, with episodic migraine being reported by 3.0% of the patients. Chronic migraine or probable chronic migraine was reported by 3.5% of the patients. The conclusions of this study included that in people above 65 years of age seeking medical care for memory and cognitive decline, headache is a common problem impacting health and requiring treatment [7].

An important point that has been highlighted in literature is that after menopause, female predominance declines [8••, 9]. In a separate study [10] that evaluated the charts of 239 patients with migraine who were older than 64 years of age, it was concluded that the frequency of migraine for these older patients was higher if the first onset of their migraine episodes occurred before they were 18 years of age. In addition, if migraine onset started prior to the age of 18, one in three of those patients would have migraine that persisted into older age [10].

## Diagnostic Approaches and Considerations

In seniors, headache is most likely to be primary [11]. Within this population, the diagnosis of migraine requires ruling out secondary headache and specific attention should be paid to side effects of medications, their interactions, and comorbidities. In comparison to patients of younger ages, older adults are about 12 times more likely to have secondary headache as the etiology of new onset headache [12].

Published studies [8••, 13] have shown that the presentation of migraine in older adults can be divergent. In a Framingham cohort study of 2110 enrolled subjects, aura without associated headache was reported in 1–2% of older adults [8••, 13]. Many treatments for migraine can affect the blood vessels, such as the vasoconstrictive action of triptans. This makes it even more important to differentiate aura of migraine from stroke and in these cases, imaging of the brain should be considered.

Checking blood sedimentation rates should always be considered in older adults when they present with new headache or report changes in their headache symptoms. Patients with giant cell arteritis (GCA) may present to seek medical attention in equal proportion to migraine [14••, 15]. Giant cell arteritis is usually seen in people after 50 years of age and is more common in women [16, 17]. Treatments for GCA include steroids, methotrexate, and tocilizumab.

Checking additional blood laboratory values may also reveal different causes for new headache symptoms. There are some reports that hypothyroidism can be an underlying etiology of headache [18].

Cardiac cephalalgia is another consideration in the older adult population. Patients might present without chest pain. Headache can be triggered by exercise and relieved by nitroglycerin. A neurovascular work up should be immediately initiated, and the underlying cardiac condition treated [14••, 19].

While diagnosing migraine in older adults, ocular (glaucoma) and orbital pathology (cavernous–carotid fistula) also need to be ruled out [14••, 20].

For headache that worsens at nighttime, consideration should be given to brain imaging to help rule out brain tumors or mass lesions. Measurement of spino-cerebral fluid pressure should also be considered [14••]. Sleep disorders such as obstructive sleep apnea should be ruled out as a cause of nighttime and morning headache exacerbation.

In this regard, one published study [21] evaluated headache in the morning as a symptom of obstructive sleep apnea. This was a retrospective review of 1131 patients who underwent polysomnography for suspected obstructive sleep apnea. Morning headache was reported in 29% of participants [21]. This points to the importance of addressing obstructive sleep apnea in an elderly population reporting headache.

Another very important consideration is to address hypertension in older adults, as research suggests a multifactorial link between headache and hypertension [22]. Acute severe hypertension is a known cause of headache in the older adult population [14••].

Headache can be present in 27 to 73% of patients who are on hemodialysis. Avoiding caffeine can prevent hemodialysis headache in some cases by eliminating the symptoms of caffeine withdrawal that occur with hemodialysis [23].

Reactivation of dormant varicella zoster virus (VZV) in the elderly can lead to post-herpetic neuralgia [24]. Age is a risk factor for the progression of an acute herpes zoster episode to post-herpetic neuralgia [24]. Before vaccines, up to 90% of US adults were tested seropositive for VZV (varicella zoster virus) so headache presentation with VZV reactivation is not uncommon.

Headache associated with central nervous system infections, such as acute meningitis, require immediate diagnosis (most often by lumbar puncture and analysis of the CSF fluid) with subsequent treatment of the infection [20].

## Primary Headache Factors

In older adults, primary headache should be considered only after secondary headache is ruled out [25]. A retrospective study evaluating adverse drug reactions in a patient population of 65 years and older showed that 74.8% reports of headache occurred in women. In that study, 46.5% of adverse drug reactions were considered serious. The most frequent drugs associated with headache as a side effect were anti-virals, anti-depressants, anti-dyslipidemic agents, and central nervous system-acting analgesics [26]. Multiple other medications such as estrogens, nitrates, and levodopa may also induce headache [27].

A special consideration of the differential diagnosis of migraine and other primary headache disorders includes hypnic headache. It is hypothesized that an age-related dysfunction of the suprachiasmatic nucleus of the hypothalamus plays a role, and this is the reason that hypnic headache mostly affects older adults. [28–31]. First-line therapy for hypnic headache is caffeine. Lithium or indomethacin may be other choices, but these medications are more frequently discontinued due to side effects [32].

## Older Adults Can Have Different Migraine Symptoms

Research shows that older adults can have different migraine symptomatology [14••]. Migraine in seniors is more often bilateral. Migraine in older adults can present without sensitivity to light and sound and with less cranial autonomic symptoms [33, 34].

Recent literature has addressed the etiology of changes in clinical symptoms of migraine at different ages, hypothesizing a connection of hypothalamic areas with different brainstem areas, and including central parasympathetic areas changes throughout life that leads to changes in clinical manifestations of migraine [34].

In 1980, C. Miller Fisher described transient neurologic symptoms in older adults which can mimic transient ischemic attacks. These symptoms were labeled “late-life migraine accompaniments”. The most common symptoms of these episodes are visual changes, then sensory, aphasia, and motor changes. “Late-life migraine accompaniments” can be applied in the literature to the description of aura symptoms without headache or as new aura with headache, which many older adults can experience. Differential diagnostic protocols can include brain imaging and electroencephalography [35].

## Episodic Migraine Treatment Considerations for Older Adults

In older adults, medication interactions can have a very significant role in the etiology of episodic migraine. In one study, it was reported that 89.1% of patients in the study fulfilled at least one of the Beers criteria [36, 37•].

A 2019 update [37•] to the Beers criteria included reporting the use of opioids in older adults and this requires special attention. In that study, it was reported the largest relative increase in opioid use was between two age groups; in those people 55–64 years of age and people 65 years and older [37•]. In addition to headache, opioid analgesics can lead to cognitive decline and sedation [38].

The use of acetaminophen to treat pain in the elderly should prompt the caregiver to monitor liver functions [39, 40]. Nonsteroidal anti-inflammatory medications should also be used with caution for acute pain management in an elderly population, and these medications should only be given if there is a close monitoring of kidney and liver function, as well as following any harmful effects on the gastrointestinal and cardiovascular systems [40].

The use of triptans and ergots can be limited by their vasoconstrictive effects in older adults.

CGRP blocking agents can be used in older adults, when appropriate.

New research offers more options for seniors, including acute non-opioid treatments with lasmiditan [41], ubrogepant [42], and rimegepant [43]. We are also in a new era of headache research when older adults start be included in clinical trials.

Neuroleptics and anti-emetics should be used with caution in older adults as these medications can lead to drug-induced Parkinsonism and sedation [44].

Tricyclic antidepressants use can lead to cognitive impairment, arrhythmia and urinary retention, weight changes, and disruption of sleep [45]. Valproic acid might induce liver enzyme disorders and common side effects of valproic acid include gastrointestinal symptoms and tremor [46].

The antiplatelet effects of verapamil can lead to gastrointestinal bleeding [47].

Candesartan can be often used in an older adult population for headache preventive treatment safely [39].

The medications mentioned above are just a few examples of why it is important, especially in the elderly, to start medications at a very low dose and then titrate up slowly while monitoring for side effects.

Onabotulinum toxin (Botox®) is not recommended for episodic migraine treatment. However, neuromodulation devices in patients without pacemakers, as well as nerve blocks, can be used as appropriate in patients with episodic migraine [36].

Finally, lifestyle modifications and behavioral modalities (for example, mindfulness) are especially important in this population and should receive serious consideration [48].

## Conclusion

The percentage of people living past the age of 60 has dramatically expanded in the twenty-first century. As migraine has an annual prevalence of 10% in these older adults, caregivers will be faced with the challenge of treating headache in these individuals who often present with a host of comorbidities that are being treated by multiple medications. However, with an appropriate diagnostic work up and a careful consideration of a treatment plan, headache in these older patients can be managed in a manner that returns them to a better functioning and higher quality of life.
